# Female Groin Hernia Repairs in the Swedish Hernia Register 1992–2022: A Review With Updates

**DOI:** 10.3389/jaws.2023.11759

**Published:** 2023-09-27

**Authors:** Ursula Dahlstrand, Maria Melkemichel, Johanna Österberg, Agneta Montgomery, Hanna de la Croix

**Affiliations:** ^1^ Department of Clinical Science, Intervention and Technology, Karolinska Institutet, Stockholm, Sweden; ^2^ Department of Surgery, Enköping Hospital, Enköping, Sweden; ^3^ Department of Clinical Science and Education, Södersjukhuset, Karolinska Institutet, Stockholm, Sweden; ^4^ Department of Surgery, Södertälje Hospital, Södertälje, Sweden; ^5^ Department of Surgery, Mora Hospital, Mora, Sweden; ^6^ Department of Clinical Sciences, Malmö, Lund University, Lund, Sweden; ^7^ Department of Surgery, Institute of Clinical Sciences, Sahlgrenska Academy, University of Gothenburg, Gothenburg, Sweden; ^8^ Department of Surgery, Region Västra Götaland, Sahlgrenska University Hospital, Gothenburg, Sweden

**Keywords:** women, inguinal hernia, femoral hernia, register study, gender differences

## Abstract

**Introduction:** Groin hernias in women is much less common than in men; it constitutes only 9% of all groin hernia operations. Historically, studies have been performed on men and the results applied to both genders. However, prospectively registered operations within national registers have contributed to new knowledge regarding groin hernias in women. The aim of this paper was to investigate and present a body of literature based upon the Swedish Hernia Register together with recent data from the register’s annual report.

**Patients and Methods:** PubMed and Embase were searched for studies based on the Swedish Hernia Register between 1992 and 2023. Based on the initial reading of abstracts, studies that presented results separately for women were selected and read. Recent data were acquired from the 2022 annual report of the Swedish Hernia Register.

**Results:** A total of 73 studies of interest were identified. Of these, 52 included women, but only 19 presented separate results for women. Four themes emerged and were analysed further: emergency surgery and mortality, femoral hernias, the risk of reoperation for recurrence, and chronic pain following female groin hernia repairs.

**Discussion:** Studies from the Swedish Hernia Register clearly describe that both the presentation of hernias and outcomes after repair differ significantly between the two genders. The differences that have been identified over the years have been incorporated into the national guidelines. Register data indicates that the guidelines have been implemented and are fairly well adhered to. As a result, significant improvements in outcomes regarding recurrences have been made for women with groin hernias in Sweden.

## Introduction

A groin hernia is a common condition, and several million groin hernia repairs are performed annually worldwide. There is a vast body of scientific evidence regarding treatment and outcomes.

While all of this is true for men, groin hernias in women are a much less common condition. Only 9% of the groin hernia repairs registered in the nationwide Swedish Hernia Register (SHR) are performed on women [1]. No randomised controlled trials have been performed specifically on women, and other prospective studies on female groin hernia surgery are sparse in the current literature [2].

Following the more wide-spread adoption of mesh techniques, such as the Lichtenstein technique in the 1990s, the risk of reoperation for recurrence became markedly reduced in men [3, 4]. This tension-free open mesh hernioplasty method of repair was also introduced for women.

In 1992, Swedish surgeons started to register groin hernia repairs in the SHR, and in time, a unique nationwide database of prospectively registered operations emerged that now offers opportunities to study women with groin hernia repairs in more detail [5]. Over the years, the different aspects of groin hernia repair in women that have received increasing study are mortality, outcome following femoral hernia repairs, the risk of reoperation for recurrence, hernia anatomy and the risk of chronic pain following groin hernia repair.

The aim of this review was to summarise 31 years of groin hernia repair in women using the latest national report from the SHR and to review previous studies based on data from the SHR pertaining to groin hernia in women with the intention of highlighting the foremost insights regarding groin hernia repair in women.

## Material and Methods

### The Swedish Hernia Register

The SHR is a non-mandatory quality register that is concerned with groin hernia repair in adults. It was established in 1992 with only eight hospitals being aligned in the beginning. The objective was to study and analyse groin hernia repairs in Sweden and stimulate improvement at participating units by comparing local outcomes with those from the rest of the nation. Today, some 90 units participate, and more than 90% of all groin hernia operations performed in Sweden are registered with the SHR, which has created a national database of more than 400,000 operations. Procedures are recorded prospectively, and patients are identified by their personal identity number, which is unique for each citizen in Sweden [6]. Patients are followed from operation until reoperation, death or emigration. SHR is linked with the Swedish population register, which allows more accurate follow-up times.

Many variables have been unchanged over the years, but others have been added, and a few have been discarded. Data registered includes patient and hernia characteristics (e.g., BMI, smoking status, comorbidity, hernia anatomy, size of the hernia defect, type of hernia, etc.), technical details (method of repair and anaesthesia, use of mesh, type of mesh, fixation) and complications occurring within 30 days (including severity and need for readmission or reoperation). Reoperations for recurrence, infection or pain are included.

In addition, between 2012 and 2018, a pain questionnaire assessing patient-reported outcome measures (PROMs) was sent out 1 year after surgery to all patients that had undergone a groin hernia repair in a unit participating in the SHR. The distributed questions were collected with a 70% response rate, which is unusual for national cohorts. The short-form PROMs questionnaire included one question concerning patient satisfaction and one from the Inguinal Pain Questionnaire (IPQ) assessing chronic pain in the groin following a groin hernia repair [7]. A short-form questionnaire has been shown to be exchangeable to the longer original version of IPQ [8]. The intensity of the pain is assessed using a 7-step fixed-rate scale with steps operationally linked to pain behaviour or pain descriptors. Responses that indicate that the pain cannot be ignored and affects daily activities are regarded as moderate to severe pain. The PROM pain question and possible answers are provided in Supplementary Appendix S1.

The SHR employs a standardised annual validation of registered data, where 10% of the aligned units are audited by external evaluators. The validity of the register has been studied with a finding of 98% correct variables and a 97% cover rate for procedures in the participating units, making it a register with high validity [9].

### Study Design

A review was conducted on previously published studies based upon data from the Swedish Hernia Register between 1 January 1992 and 30 April 2023. PubMed and Embase were searched for studies based on data from the Swedish Hernia Register, applying the search string “‘hernia’ AND (‘Swedish Hernia Register’ OR ‘Swedish Hernia Registry’)”. Conference abstracts were disregarded. These searches were checked against a list of studies kept by the SHR. Any additional studies found there were also identified and accessed by online search. Abstracts were read by all authors. Only studies specifying results for the female gender were included in the review.

### Study Population

Recent data and figures were adopted from the 2022 annual report, and information from the SHR website [1, 10]. Data for 1992–2022 is presented because of its historical value. Data from a more modern period, 2012–2022, is presented separately, also coinciding fairly well with the introduction of national treatment recommendations for groin hernia from the National Board of Health and Welfare in 2011 [11].

## Results

In total, 73 studies based on the Swedish Hernia Register, between 1 January 1992 and 30 April 2023, were published in peer-reviewed journals. Two were reviews, and one was a study protocol. Fifty-two of the original studies included women, whereas 19 of them presented results separately for the genders and are included in this article (see Supplementary Appendix S2). Four important topics concerning female groin hernia repairs were deducted and presented below. These themes of interest are emergency surgery and mortality, femoral hernia, risk of reoperation for recurrence and chronic pain following female groin hernia repairs. A description of the current data from the SHR regarding female groin hernia repairs is also presented and illustrated in tables and figures.

### Current Data Regarding Female Groin Hernia Repairs in the SHR

**FIGURE 1:**
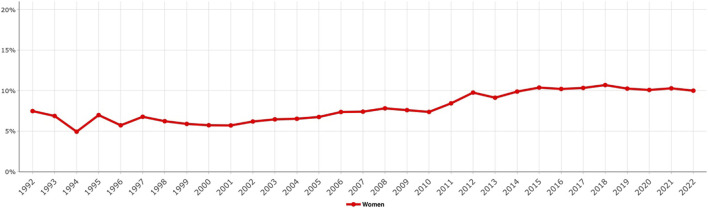
Proportion of female groin hernia repairs in the Swedish Hernia Register, 1992–2022.

**FIGURE 2:**
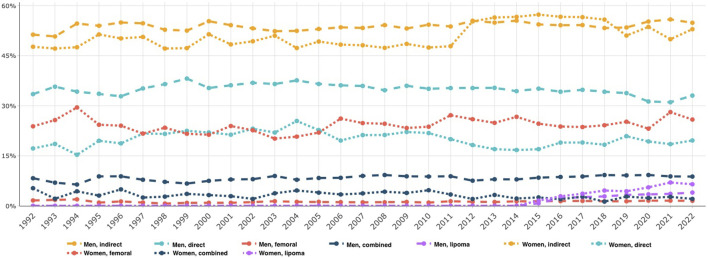
Proportion of hernia anatomy in all groin hernia repairs in women compared to men over time, 1992–2022.

**FIGURE 3:**
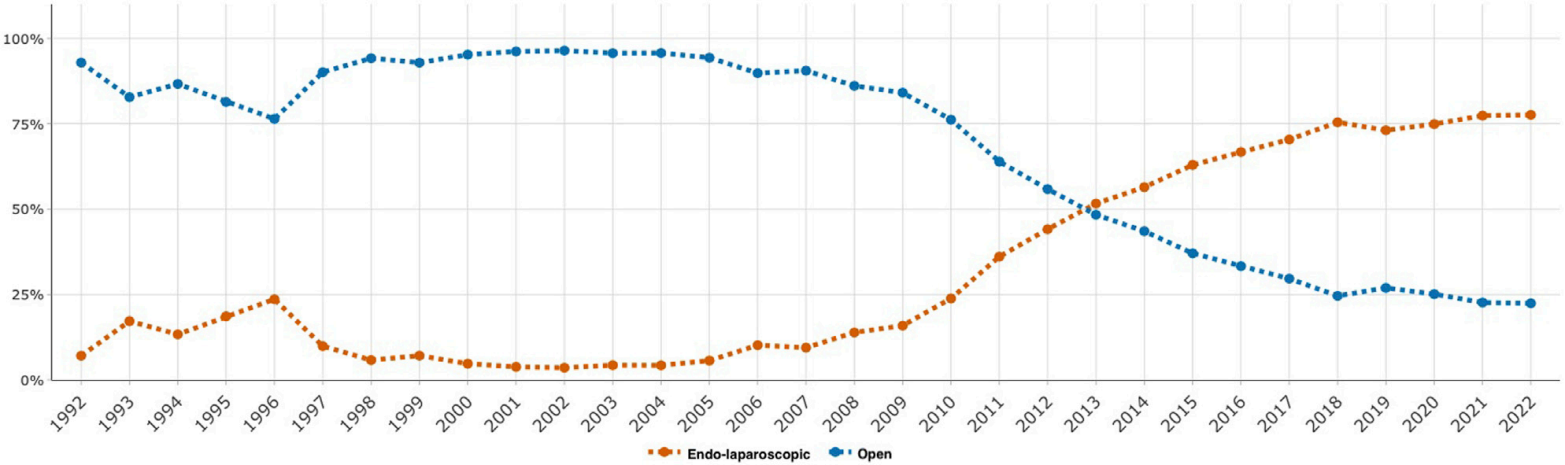
Proportion of groin hernia repairs in women for endo-laparoscopic versus open surgery over time, 1992–2022.

**FIGURE 4:**
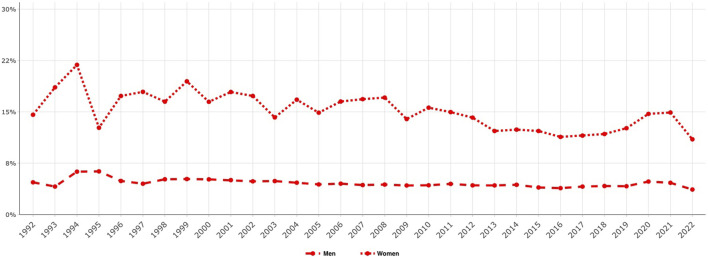
Proportion of emergency groin hernia repairs over time, women compared to men, 1992–2022.

**FIGURE 5:**
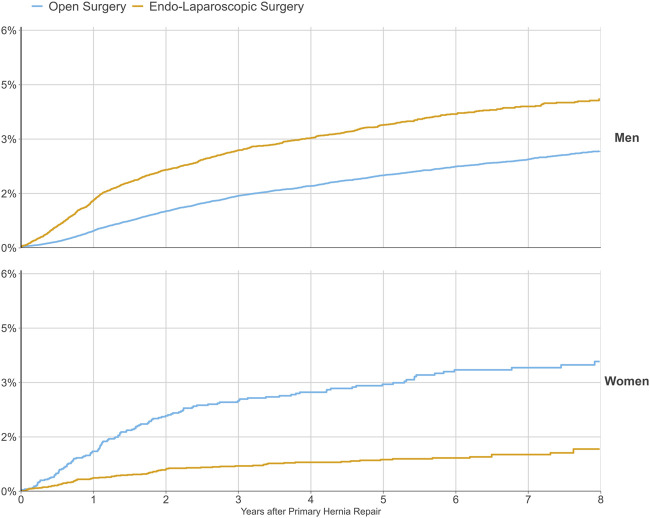
Risk of reoperation for recurrence following a primary groin hernia repair with different surgical methods of repair between 2012 and 2022, demonstrated separately for women and men.

In Sweden, approximately 16,000 groin hernia operations are registered annually in the SHR, except for 2020 due to the COVID-19 pandemic, during which the amount decreased to 12,689 operations [10]. In 2021, the number of registered operations increased again. Gender data has been reported in the SHR since the beginning. Cumulative demographic characteristics of female groin hernia repairs in the SHR in the years 1992–2022 and 2012–2022 are presented in Tables 1, 2. Developments and trends in female groin hernia surgery in the SHR can also be followed by looking at changes over time between 1992 and 2022 in Figures 1–5.

**TABLE 1:** Female groin hernia repairs in the Swedish Hernia Register, 1992–2022, in total and divided by surgical technique into open and endo-laparoscopic surgery.

	Open surgery	Endo-laparoscopic surgery	All female repairs
Patients	19,514 (63.6)	11,030 (35.9)	30,699
Age, years	63.9	55.5	61.2
Hernia repairs	21,414 (60.1)	13,547 (38.5)	35,179
Reoperation for recurrence	1,041 (87.1)	137 (11.5)	1,195
Type of Hernia			
Primary	20,026 (60.7)	12,837 (38.9)	33,001
Recurrent	1,353 (65.5)	699 (33.8)	2,066
Type of Hernia			
Unilateral	20,861 (66.5)	10,292 (32.8)	31,362
Bilateral	553 (14.5)	3,255 (85.3)	3,817
Emergency repairs	4,390 (86.9)	619 (12.3)	5,052

Data are in numbers with percentages in parentheses. Due to missing data in some variables during the earlier years of the SHR the sum of percentages does not always equal 100. Age is presented in median.

**TABLE 2:** Female groin hernia repairs in the Swedish Hernia Register, 2012–2022, in total and divided by surgical technique into open and endo-laparoscopic surgery.

	Open surgery	Endo-laparoscopic surgery	All female repairs
Patients	5,177 (34.8)	9,682 (65.2)	14,859
Age, years	68.8	59.1	62.6
Hernia repairs	5,895 (33.3)	11,764 (66.7)	17,659
Reoperation for recurrence	183 (62.9)	108 (37.1)	291
Type of Hernia			
Primary	5,483 (32.6)	11,290 (67.4)	16,773
Recurrent	375 (44.7)	463 (55.3)	838
Type of Hernia			
Unilateral	5,725 (38.8)	9,044 (61.2)	14,769
Bilateral	164 (5.7)	2,723 (94.3)	2,887
Emergency repairs	1,648 (74.6)	561 (25.4)	2,209

Data are in numbers with percentages in parentheses. Age is presented in median.

#### The Basic Characteristics of Female Groin Hernia Repairs

The number of registered groin hernia repairs in women from 1992 to 2022 was 35,179 (Table 1). The proportion of female groin hernia repairs has increased over time from around 6% of all groin hernia repairs in the early 2000s to approximately 10% in the past 8 years (Figure 1). Demographics for female groin hernia patients during the different time periods, which are classified in terms of open or endo-laparoscopic surgery, are presented in Tables 1, 2. The median age of women is consistent over time. Women who underwent endo-laparoscopic surgery were younger than women who underwent open surgery (Tables 1, 2).

In Figure 2, the hernia anatomy, as recorded by surgeons at participating units in the SHR, demonstrates that femoral hernias made up 28.6% of women’s hernias in 2021. This can be compared to a much lower proportion of femoral hernias in men (approximately 1%). Lateral inguinal hernias are still the most common hernia type registered by the SHR for women who undergo groin hernia surgery.

Rosemar et al. investigated the association between BMI and groin hernia surgery in 2010 [12]. They found a marked overrepresentation of females among patients with a BMI < 20 kg/m^2^ (29.3% as compared to 7.7% in the entire study cohort of 49,094 patients). Femoral hernias were over four times as common in this group of patients, and the gender ratio (male:female) for femoral hernias in the group was 1:4.4 rather than the 1:1.9 for the entire study cohort. Another study that used data from the SHR investigated whether waist circumference was a better predictor for groin hernia or surgery for groin hernia than BMI but did not find a difference between the two measures of body composition in either men or women [13].

A study that investigated tobacco as a risk factor for the repair of groin hernias did not establish an association in women; however, the possibility of drawing firm conclusions was limited due to the fact that only patients accepted for surgery were categorised as hernia patients [14].

#### The Surgical Method of Repair

The proportion of female groin hernia repairs performed using the endo-laparoscopic technique increased from 38.6% to 66.7% over the study period (Tables 1, 2). This change in the surgical method of repair in women over time is also illustrated in Figure 3, which indicates an increase over time of endo-laparoscopic repairs compared to open repairs beginning in 2010. Endo-laparoscopic repairs constituted 77.6% of all repairs in women in 2022 (Figure 3).

### Emergency Hernia Repair, Mortality and Morbidity

The cumulative percentage of women who underwent emergency repair for their groin hernias was almost triple that of men (14.4% vs. 5.3%) during the 1992–2022 period. The change over time is illustrated in Figure 4. Studies from the SHR have consistently shown that groin hernia repairs in women more often are carried out as an emergency then in men [15, 16].

Emergency surgery is associated with significantly increased mortality [17, 18]. In 2007, data from the SHR was merged with that from the death register and the standardised mortality ratio was calculated using death statistics in Sweden. 107,838 hernia repairs, including 8,182 repairs in women, were analysed. After emergency surgery, women had a higher risk of mortality than the background population, with a standardised mortality ratio of 20.49 (95% CI 13.26–30.25) if bowel resection was undertaken and one of 5.36 (95% CI 3.47–7.92) if no bowel resection was needed [18]. When the medical records of patients who had died within 30 days of hernia repair were examined, only 37% of patients with signs of bowel obstruction had had a documented groin examination upon admittance, and women were significantly less likely to undergo a groin examination compared to men [19].

A protective impact on risk for postoperative complications within 30 days of operation was seen in a study in 2012 [20]. When adverse events were studied later, and merged data from the SHR with data from the national patient register, the male gender was found to be associated with an increased risk of both cardiovascular events and intraoperative complications [21]. A study from 2002 found that although women had neither more registered nor more self-perceived adverse events, they sought healthcare advice regarding their perceived complications more often (in 75% of cases versus 48% for men, *p* = 0.003) in a study cohort from 2002 [22]. A tendency, although not statistically significant, for women to file damage claims more often (OR 1.62, 95% CI 0.98–2.71, *p* = 0.06) after groin hernia surgery was found by Nordin et al., while they were investigating damage claims between 2008 and 2010 [23]. Women (and men) who were lean (BMI < 20 kg/m^2^) or overweight (BMI > 25 kg/m^2^) had an increased risk of postoperative complications within 30 days of a groin hernia repair [12].

### Femoral Hernia Repair

The first study using SHR data that focused on femoral hernias was published in 1999, but it included few comparisons between men and women [24]. A larger cohort of femoral hernia repairs was studied by Dahlstrand et al., who analysed 3,980 femoral repairs with a focus on recurrence, mortality and emergency operations [16]. The study demonstrated that 22.8% of groin hernia procedures in women were due to femoral hernias, whereas the corresponding proportion for men was 1.1%. Emergency procedures were more common in women, constituting 40.6% of their repairs (22.8% for men, *p* < 0.001). A study where 442 femoral hernia patients with emergency repairs retrospectively reported whether they had sought medical advice regarding their hernia and/or known about it prior to their surgery indicated that only 46.7% of the patients were aware of their hernias, while 31.3% denied symptoms from the groin more than 2 weeks before surgery. There were no differences between men and women in terms of symptoms [25].

### Risk of Reoperation and Method of Repair

Each hernia operation is followed in the SHR until either reoperation of the same groin or the patient’s death. This allows the probability of reoperation due to recurrence to be calculated as a function of the time after a hernia operation. Before 2005, gender had not been assessed as a risk factor for recurrence in studies from the SHR. In 2005, 6,895 prospectively registered groin hernia operations in women were studied specifically. Koch et al. found that women had a significantly increased risk of having a reoperation because of a recurrence (HR 1.30, 95% CI 1.13–1.49) [15]. They also found that Lichtenstein, the repair method with the lowest risk of reoperation in men, was associated with the highest risk for reoperation in women. Furthermore, femoral hernia was diagnosed in 41.6% of reoperations after hernias originally classified as inguinal hernias. A more recent study of more than 17,000 repairs in women confirmed the results and reported that by far, endo-laparoscopic repair was the method associated with the least risk for reoperation in women but, doubled the risk for reoperation due to recurrence in men [26]. Koch et al. suggested that surgeons missed femoral hernias when open anterior mesh techniques were used. Thus, the 2011 national guidelines recommended a laparoscopic posterior mesh repair in all groin hernias in women due to the superior ability to visualise all groin hernia orifices during the procedure. The method of choice has changed for women in Sweden since then (Figure 3).

Today, the female gender is no longer associated with an increased risk of reoperation in Sweden. Figure 5 indicates the cumulative risk of reoperation for recurrence following a primary groin hernia repair with open versus endo-laparoscopic surgery. In Cox regression analysis regarding reoperation for recurrence following open or endo-laparoscopic surgery for primary groin hernia repair 2013–2022, stratified by gender, endo-laparoscopic repair was associated with a lower risk for reoperation than open repair for women [hazard ratio (HR) 0.303; 95% CI 0.232–0.396; *p* < 0.001] but a higher risk for men (HR 1.728; 95% CI 1.601–1.864; *p* < 0.001) [10]. The difference in the effectiveness of totally extra-peritoneal (TEP) groin hernia repair for the different genders was also demonstrated in a study of TEP procedures registered in the SHR 2005–2013 [27]. The authors found that women had a lower risk for reoperation due to recurrence (HR 0.39, 95% CI 0.26–0.59) compared to men.

### Chronic Pain

One of the most important adverse events following groin hernia surgery is chronic pain. A total of 4,021 women with unilateral primary groin hernia repair answered the 1 year follow-up questionnaire for patient-reported outcomes that was sent out by the SHR between 2012 and 2018. A significantly larger proportion of women than men reported suffering from chronic pain; 18.4% of women versus 15.2% of men [28]. In the same study, women also reported severe pain more frequently. Moreover, TEP groin hernia repair was associated with less chronic pain in men but not in women. However, a similar trend was indicated for women. Bjurström et al. investigated 955 women and 1,129 men with repairs who had completed the PROM during the same time period [29]. The authors found that the differences in pain between genders described at 1 year after surgery were still present 5 years after surgery when assessed with the Brief Pain Inventory [30]. Reoperation due to pain after groin hernia repair is rare, with a frequency of 0.13% in the SHR, but women have been found to be at greater risk (HR 2.13, 95% CI 1.41–3.21) [31].

The association between sleep disturbances and chronic pain after groin hernia repair has been investigated, with a specific focus on differences between the genders [29]. Retrospectively, women reported persistent sleep problems prior to surgery more often than men. Preoperative sleep difficulties were a significant risk factor for chronic pain both 1 year after surgery and in the long-term follow-up.

Chronic pain has also been studied for the subpopulation with femoral hernia repairs. Pain was demonstrated to be as common as after inguinal repairs in a study that investigated 1,461 patients who completed the IPQ at a median follow-up time of 4.7 years after surgery. Women constituted 72% of the 1,461 respondents; no differences between the genders were seen in terms of pain, with 5.5% of patients reporting pain of at least moderately severe intensity [32].

## Discussion

While groin hernias in women are infrequent, information regarding more than 35,000 repairs on females has now been gathered in the SHR over 30 years. The collected data encompasses the vast majority of repairs in the country and constitutes an unparalleled cohort. In this article, we demonstrate how results based upon this cohort have changed the surgical management of groin hernias in women, significantly improving the outcome after surgery.

During the first decade of the SHR’s history, female repairs were included in the results presented, but no analyses taking gender into account were performed. This was not unique to Sweden. In fact, there is an absolute paucity of scientific reports regarding groin hernia in women prior to the early 2000s. Shortly after the study by Koch et al. was published in 2005, the Danish Hernia Database (DHD) published data that concentrated on outcomes for women and showed a higher risk for reoperation among women due to recurrence compared to men [15, 33]. From then on, gender has been included as an independent factor or a variable of stratification in virtually all studies based on the data from the SHR that include both genders as shown in this study.

The results from these two studies (which showed a surprising number of femoral recurrences in women) together with the evidence that preperitoneal mesh techniques were superior in femoral hernias formed the foundation for the Swedish national guidelines on groin hernia repair which were issued in 2011. For the first time, a preperitoneal technique was recommended for women, and endo-laparoscopic techniques were preferred [11, 16]. The recommendations were also communicated in a state-of-the-art lecture during the 2011 national conference of the Swedish Surgical Society. The superiority of endo-laparoscopic repairs for femoral hernias has been confirmed in a Danish register study [34]. Since occult femoral hernias have been proven to be considerably more common in women and the endo-laparoscopic techniques have a clear advantage in being able to diagnose and treat possible femoral hernias, the scales have tipped in favour of such techniques [35, 36]. More recent studies in Sweden have confirmed that endo-laparoscopic repairs are associated with a lower risk of recurrence in women [26, 27]. In 2019, a study demonstrated the same pattern in Denmark. Hernias that are repaired with a laparoscopic mesh technique in women are much less prone to recur, regardless of whether the primary hernia is inguinal or femoral [37]. The international guidelines now present a strong recommendation that endo-laparoscopic mesh repair is the recommended method of choice for women [2]. From the annual report of the SHR and the results reported in this study, it can be deduced that these guidelines are being followed, and the results in terms of recurrences in women have greatly improved as a result of that.

In terms of mortality risk after emergency hernia repair in studies from the SHR, similar results have been reported from the Danish Hernia Database and the Herniamed register. The distinct increase in mortality, especially when bowel resection is needed, is now well established [38, 39]. Regretfully, both a Swedish study and a Danish study have shown that delays in diagnosis as well as logistics cause delays in surgical treatment [19, 40].

The female gender has previously been described as a risk factor for chronic post-operative pain [41, 42]. SHR-based studies have come to the same conclusion [28, 29, 31]. The reasons for this are not known, and the difference persists even though a larger proportion of women than men have minimally invasive procedures. This is an evident area for further improvement of groin hernia treatment in women. There are limitations to register studies. There is a limitation to the number of variables that can reasonably be included in a register for routine care. In the SHR data regarding history of pain medication is not available for analyses, for example. Neither does it to date include information regarding preoperative pain, although there are plans for including it in the near future.

The case of groin hernia in women illustrates the need for different types of scientific studies in evidence-based medicine. Even with a very common affliction, like a groin hernia, it is possible that subgroups do not follow the same pattern as the larger group of patients. One of the great strengths of quality registers with a high cover rate is the possibility of studying subpopulations or less common outcomes and providing knowledge in these areas. It has been proven that both the quality of outcomes and the cost-effectiveness of groin hernia repair have been improved in Sweden thanks to the register, especially among women who undergo groin hernia repairs.

## Conclusion

Women constitute a subgroup of groin hernia patients. Knowledge gained from the large quality registers has pointed out gender inequalities and has been instrumental in providing guidelines tailored for women. Data from the Swedish Hernia Register demonstrates that results regarding recurrence have improved for women as the guidelines have been implemented. Today, women who are operated with an endo-laparoscopic repair for a groin hernia in Sweden have a lower risk of reoperation due to recurrence than men.

## Data Availability

The original contributions presented in the study are included in the article/Supplementary Material, further inquiries can be directed to the corresponding author.
