# Supplementary material for: Female Groin Hernia Repairs in the Swedish Hernia Register 1992–2022: A Review With Updates
Source: J Abdom Wall Surg. 2023 Sep 27;2:11759. doi: 10.3389/jaws.2023.11759 (PMC10831639; doi:10.3389/jaws.2023.11759)
Supplement: Supplementary file 1 [file DataSheet2.PDF]

Peer-reviewed articles based on data from the Swedish Hernia Register (SHR) 1992-2022, reporting results for women specifically. Identified through PubMed and Embase search, and by information from the SHR.

**Author Names**	**Title**	**Source title**	**Publication Year**	**Outcome**	**Specifies outcome for women**	**Number of women included**
Hemberg A., Montgomery A., Holmberg H., Nordin P.	Waist Circumference is not Superior to Body Mass Index in Predicting Groin Hernia Repair in Either Men or Women	World journal of surgery	2022	risk factors for groin hernia	yes	56597 (whereof 329 with hernia)
Jakobsson E., Lundström K.-J., Holmberg H., De La Croix H., Nordin P.	Chronic Pain After Groin Hernia Surgery in Women: A Patient-reported Outcome Study Based on Data From the Swedish Hernia Register	Annals of Surgery	2022	chronic pain	yes	4021
Bjurström M., Irwin M., Chen D., Smith M., Montgomery a.	Sex Differences, Sleep Disturbances and Risk of Persistent Pain Associated With Groin Hernia Surgery: A Nationwide Register-Based Cohort Study	The Journal of Pain	2021	chronic pain	yes	955
Melkemichel M., Bringman S., Widhe B.	Lower recurrence rate with heavyweight mesh compared to lightweight mesh in laparoscopic totally extra-peritoneal (TEP) repair of groin hernia: a nationwide population-based register study	Hernia	2018	reoperation due to recurrence	yes	1691
Nilsson H., Holmberg H., Nordin P.	Groin hernia repair in women – A nationwide register study	American Journal of Surgery	2018	reoperation due to recurrence	yes	17545

Hemberg A., Holmberg H., Norberg M., Nordin P.	Tobacco use is not associated with groin hernia repair, a population-based study	Hernia	2017	risk factors for groin hernia	yes	52079 (whereof 164 with hernia)
Nordin P., Ahlberg J., Johansson H., Holmberg H., Hafström L.	Risk factors for injuries associated with damage claims following groin hernia repair	Hernia	2017	complications - damage claims	yes	4113
Nilsson H., Angerås U., Sandblom G., Nordin P.	Serious adverse events within 30 days of groin hernia surgery	Hernia	2016	intra- and postoperative complications	yes	11726
Wefer A., Gunnarsson U., Fränneby U., Sandblom G.	Patient-reported adverse events after hernia surgery and socio-economic status: A register-based cohort study	International Journal of Surgery	2016	complications- patient reported	yes	107
Hallén M., Sevonius D., Westerdahl J., Gunnarsson U., Sandblom G.	Risk factors for reoperation due to chronic groin postherniorrhaphy pain	Hernia	2015	reoperation due to recurrence	yes	13985
Dahlstrand U., Sandblom G., Wollert S., Gunnarsson U.	Limited potential for prevention of emergency surgery for femoral hernia	World journal of surgery	2014	retrospective - hernia known prior to emergency surgery	yes	1044
Lundström K.-J., Sandblom G., Smedberg S., Nordin P.	Risk factors for complications in groin hernia surgery: A national register study	Annals of Surgery	2012	postoperativ complications	yes	10805
Dahlstrand U., Sandblom G., Nordin P., Wollert S., Gunnarsson U.	Chronic pain after femoral hernia repair: A cross-sectional study	Annals of Surgery	2011	chronic pain	yes	1046
Nilsson H., Nilsson E., Angerås U., Nordin P.	Mortality after groin hernia surgery: Delay of treatment and cause of death	Hernia	2011	cause of death	yes	46

Rosemar A., Angerås U., Rosengren A., Nordin P.	Effect of body mass index on groin hernia surgery	Annals of Surgery	2010	reoperation due to recurrence, complications, mortality	yes	3760
Dahlstrand U., Wollert S., Nordin P., Sandblom G., Gunnarsson U.	Emergency femoral hernia repair: A study based on a national register	Annals of Surgery	2009	reoperation due to recurrence, mortality	yes	2490
Nilsson H., Stylianidis G., Haapamäki M., Nilsson E., Nordin P.	Mortality after groin hernia surgery	Annals of Surgery	2007	mortality	yes	6806
Koch A., Edwards A., Haapaniemi S., Nordin P., Kald A.	Prospective evaluation of 6895 groin hernia repairs in women	British Journal of Surgery	2005	reoperation due to recurrence	yes	6895
Haapaniemi S., Sandblom G., Nilsson E.	Mortality after elective and emergency surgery for inguinal and femoral hernia	Hernia	1999	mortality	yes	364
