# Supplementary material for: Female Groin Hernia Repairs in the Swedish Hernia Register 1992–2022: A Review With Updates
Source: J Abdom Wall Surg. 2023 Sep 27;2:11759. doi: 10.3389/jaws.2023.11759 (PMC10831639; doi:10.3389/jaws.2023.11759)
Supplement: Supplementary file 2 [file DataSheet1.PDF]

Question regarding pain intensity included in the Swedish Hernia Register follow-up administered one year after surgery to patients with a groin hernia repair, 2012-2017.

Estimate the worst pain you felt in the operated groin during this past week

No pain	☐
Pain present but can easily be ignored	☐
Pain present, cannot be ignored, but does not interfere with daily activities	☐
Pain present, cannot be ignored, interferes with concentration on chores and daily activities	☐
Pain present, cannot be ignored, interferes with most activities	☐
Pain present, cannot be ignored, necessitates bed rest	☐
Pain present, cannot be ignored, prompt medical advice sought	☐
